# Effect of sintering speed and surface treatment on the flexural strength of 3Y-PSZ and 4Y-PSZ zirconia: an in vitro study

**DOI:** 10.1186/s12903-025-07318-y

**Published:** 2025-12-10

**Authors:** Moustafa Aboushelib, Mahinour Yousry

**Affiliations:** 1 Dental Materials Science Department, Faculty of Dentistry, Alexandria University, Alexandria, Egypt; 2 Fixed Prosthodontics Department, College of Dentistry, Arab Academy for Science, Technology and Maritime Transport, Alamein, Egypt

**Keywords:** Air abrasion, Ceramics, Flexural strength, Materials, Properties, Sintering, Surface, Translucent, Zirconia

## Abstract

**Backgound:**

The mechanical performance of translucent zirconia is critical to the long-term success of monolithic restorations, particularly under speed sintering and surface modification protocols. Understanding how these factors interact is essential for clinical durability.

**Aim of the study:**

This in vitro study aimed to evaluate the effect of sintering speed and surface treatments on the flexural strength of 3 mol% and 4 mol% yttria-partially stabilized zirconia (3Y-PSZ and 4Y-PSZ), before and after cyclic fatigue loading.

**Materials and methods:**

A total of 160 Bar-shaped specimens were fabricated from 3 mol% and 4 mol% yttria-partially stabilized zirconia (3Y-PSZ and 4Y-PSZ) and sintered using either conventional or speed sintering protocols. Each group received one of four surface treatments: polishing (control), airborne-particle abrasion with 50 µm glass beads, alumina abrasion, or grinding (n=20). Flexural strength was measured using a 4-point bending test. Half of the specimens were tested in their initial state, while the other half underwent 2 million cyclic loading prior to testing to assess residual strength. Weibull statistics assessed reliability; microstructure was evaluated by SEM; ANOVA with Bonferroni post hoc tests determined significance (α = 0.05).

**Results:**

Glass bead treatment significantly improved the flexural strength of 3Y-PSZ (p < 0.001), while grinding significantly reduced it in both zirconia types (p < 0.001). Speed sintering did not affect 3Y-PSZ (p = 0.10) but significantly decreased the strength of 4Y-PSZ (p < 0.001). After cyclic loading, 3Y-PSZ maintained stable strength (p = 0.082), whereas 4Y-PSZ exhibited a significant reduction (p < 0.001). The highest Weibull modulus values were recorded in polished 4Y-PSZ (12.8) and glass-bead treated 3Y-PSZ (12.9).

**Conclusions:**

3Y-PSZ is more suitable for high-stress clinical applications and can tolerate speed sintering and cyclic loading better than 4Y-PSZ, which required careful selection of sintering protocols and surface treatments to minimize reduce in its strength.

## Background

 Yttria-stabilized zirconia (YSZ) has become a material of choice in dental restorations due to its excellent mechanical strength and evolving esthetic properties [1, 2]. Over time, modifications in yttria (Y₂O₃) and alumina content have led to the development of distinct generations of zirconia to balance strength and translucency [3, 4]. The first generation, 3 mol% yttria-stabilized tetragonal zirconia polycrystals (3Y-TZP), exhibited high flexural strength (> 1350 MPa) owing to transformation toughening but lacked translucency, requiring veneering ceramics, which were prone to chipping and delamination [4–6]. To address these drawbacks, second-generation zirconia specifically 3 mol% yttria partially stabilized zirconia (3Y-PSZ) was introduced with reduced alumina content and optimized sintering protocols to improve translucency, though at the cost of reduced mechanical strength (approximately 900–1150 MPa) [3, 7]. Recent investigations have confirmed that sintering parameters, such as dwell time and heating rate, significantly affect zirconia’s microstructure and optical behavior [8]. Further developments yielded third-generation zirconia (4Y/5Y-PSZ), which increased the cubic phase content to enhance light transmission but further compromised strength, reducing flexural resistance below 900 MPa [1, 9, 10]. A recent meta-analysis confirmed that material composition, particularly yttria content and grain size, played a pivotal role in controlling optical properties across zirconia generations [11].

Sintering parameters significantly influenced the microstructure, translucency, and mechanical performance of yttria-stabilized zirconia (YSZ). Variations in sintering temperature, dwell time, and heating rate significantly affected grain size, phase distribution, and susceptibility to low-temperature degradation, all which impact both flexural strength and optical behavior [8, 12, 13]. Speed sintering has been proposed as a more efficient alternative to conventional protocols. Studies on 3Y-PSZ have shown that mechanical strength can be maintained or improved with speed sintering [14, 15]. However, highly translucent grades (4Y-, 5Y-PSZ) could exhibit grain coarsening, altered phase distribution, or increased porosity under suboptimal sintering [13, 16–18], effects that were material dependent [8, 19].

Creating a micro-roughened surface is one of the prerequisites for achieving a durable bond with zirconia. Methods such as grinding, selective infiltration etching, fusion sputtering, and airborne-particle abrasion are employed to increase micromechanical retention [20, 21]. However, surface modification techniques could compromise flexural strength. While airborne particle abrasion is widely used, it has been reported to induce microcracks, potentially compromising the integrity of the material [22–24] and its effects varied by zirconia composition and surface protocol [25].

Given the interconnected effects of sintering speed and surface conditioning, the aim of this study was to evaluate how these variables affect the flexural strength of 3Y-PSZ and 4Y-PSZ before and after cyclic loading. The null hypothesis was that sintering speed and surface treatment would not significantly affect the flexural strength of either 3 and 4 mol% yttria-partially stabilized zirconia.

## Materials and methods

### Preparation of the specimens

**Fig. 1:**
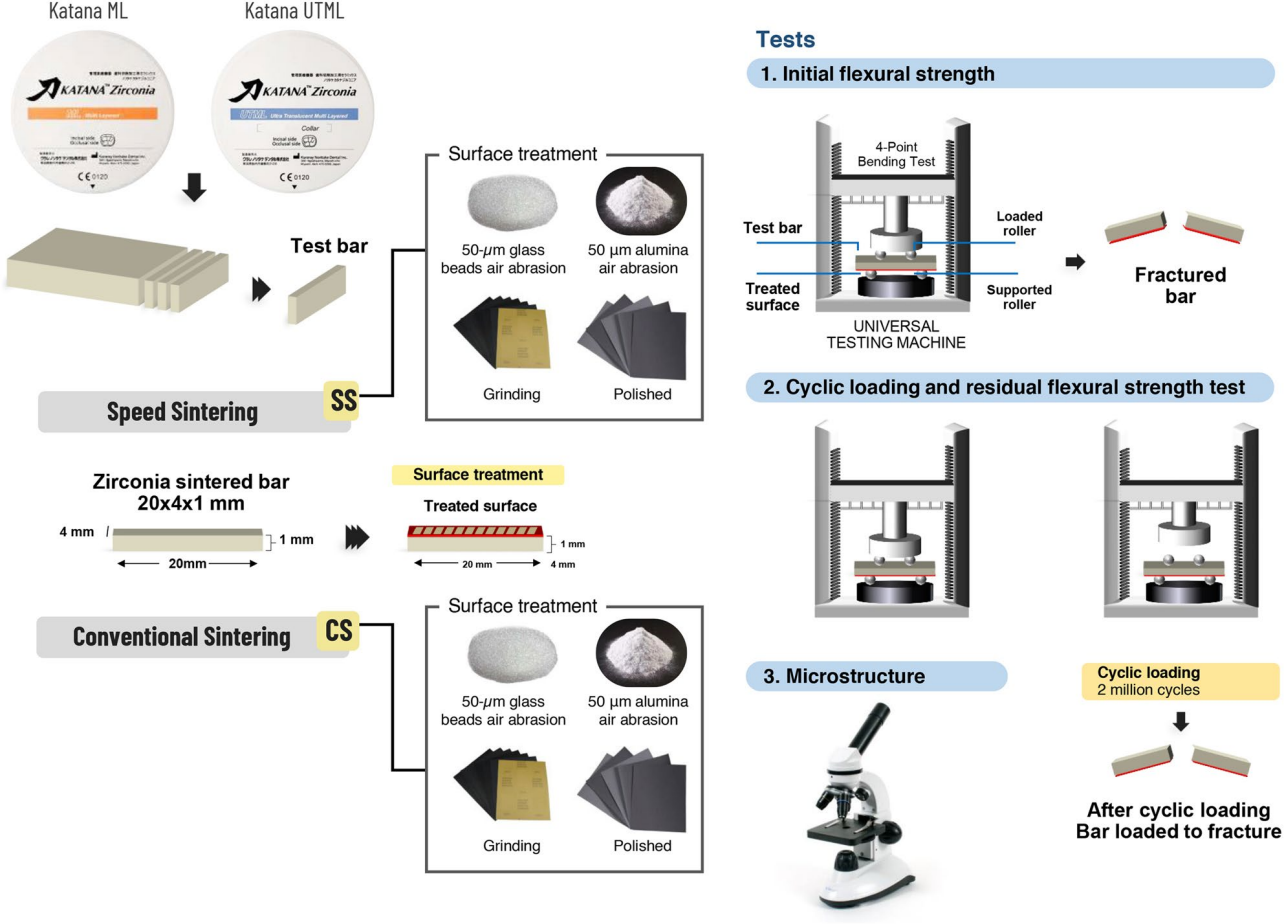
Flow chart representation of present study setup

A total of 160 bar-shaped specimens were prepared from CAD-CAM zirconia blocks of 3 mol% yttria partially stabilized zirconia (3Y-PSZ; Katana ML) and 4% mol yttria partially stabilized zirconia (4Y-PSZ; Katana STML) (*N* = 80) (Table 1; Fig. 1). The blocks were sectioned into bars using a diamond saw and a high precision cutting machine (Micracut, Metkon Instrument Ltd., Turkey). Specimens were polished with P1200, P1500, and P2500 silicon carbide abrasive sheets. Before sintering, the bars were cleaned with 78% isopropyl alcohol followed by acetone in an ultrasonic bath (iSonic Inc., Chicago, Illinois) and then air dried.

**Table 1:** Zirconia composition used in the current study

commercial zirconia name/abbreviation	Zirconia type	Manufacturer	Composition ^a^
Katana ML (Kat.ML)	(preshaded 3Y-PSZ)	Kuraray Noritake Dental Inc. (Tokyo, Japan)	90.6% ZrO_2_, 1.3% HfO2, 7.3 ± 0.2% Y_2_O_3_, 0.16% Al_2_O_3_, 0.12% other oxides
Katana STML (Kat.STML)	(preshaded 4Y-PSZ)	Kuraray Noritake Dental Inc. (Tokyo, Japan)	88% ZrO_2_, 1.5% HfO_2_, 9.7 ± 0.7% Y_2_O_3_

^a^ Data provided by the manufacturer

### Sintering of the specimens

All sintering procedures were sintered in a high-temperature dental furnace (inLab Profire, Dentsply Sirona) following manufacturer-recommended protocols for each material type. For Conventional sintering (CS), specimens were heated at a rate of 10 °C/min to 1500 °C, held for 2 h and then allowed to cool with a rate of 10 °C/min temperature decrease. For Speed sintering (SS), was performed with heating rate of 35 °C/min heating, till reached 1560 °C, held for 30 min, then cooled with a rate of 45 °C/min (Table 2) [26]. These parameters were selected to reflect clinically relevant CAD/CAM protocols for each material to follow manufacturer-recommended protocols. Following sintering, final specimen dimensions (20 × 4 × 1 mm) were verified using a digital caliper (Absolute Digimatic Caliper, Mitutoyo Corp., Japan) to ensure standardization.

**Table 2:** Overview of experimental groups based on zirconia type, sintering protocol, and surface treatment. Each group (n = 20) was equally divided between specimens tested for initial flexural strength and those subjected to cyclic fatigue loading prior to testing

**Zirconia**	**Sintering protocol (Temp/Dwell/Heating -cooling rate °C/min)**	**Surface treatment (n=20)**	**Initial and residual flexural testing (n)**	**Residual flexural testing after cyclic loading (n)**
3Y-PSZ (Katana ML)	Conventional (1550 / 2 hours / 10°–10°)	Polished (control)	10	10
		Glass bead air abrasion	10	10
		Alumina air abrasion	10	10
		Grinding	10	10
	Speed (1560 / 30 minutes / 35°–45°)	Polished (control)	10	10
		Glass bead air abrasion	10	10
		Alumina air abrasion	10	10
		Grinding	10	10
4Y-PSZ (Katana STML)	Conventional (1550 / 2 hours / 10°–10°)	Polished (control)	10	10
		Glass bead air abrasion	10	10
		Alumina air abrasion	10	10
		Grinding	10	10
	Speed (1560 / 30 minutes / 35°–45°)	Polished (control)	10	10
		Glass bead air abrasion	10	10
		Alumina air abrasion	10	10
		Grinding	10	10

### Surface treatment

Each specimen (*n* = 20 per subgroup) received one of the following surface treatments (*n* = 20 per subgroup):

- Control group: a highly polished surface served as the control.
- Glass bead abrasion: air abrasion using 50 μm glass beads for 20 s at a distance of 1.27 cm and a pressure of 0.25 MPa [27].
- Alumina abrasion: air abrasion using 50 μm aluminum oxide (Al₂O₃) applied under the same conditions using a sandblaster (Airsonic; Hager Werken, Germany) [28].
- Grinding: grinding with 400-grit silicon carbide abrasive paper (3 M, St. Paul, USA) on a metallographic polishing device under water cooling with a 300 g load. The grinding procedure was applied solely as a standardized in-vitro surface preparation method to create a controlled and reproducible baseline roughness. It was not designed to replicate clinical or laboratory polishing protocols but to facilitate consistent comparison with airborne-particle abrasion and polishing treatments, as commonly employed in zirconia surface modification studies [18, 22].

### Initial flexural strength test

The edges of each tested bar were beveled, and the disks were then loaded by using a Piston-on-Three-Ball test (POB) test according to the ISO6872:2015 standard with a mechanical testing machine [29]. Half of the specimens (*n* = 10) were loaded until fracture in a 4-point flexural strength test using a universal testing machine (Instron 1186, Instron, Canton, USA), with the treated surface placed in tension. Each bar was positioned on two supporting rods, with a gap of 16 mm. The crosshead moved at a speed of 1 mm/min, and the load was applied till it fractured [29]. The maximum load (N) was recorded, and the flexure strength (MPa) was determined using the given equation: 𝜎 = 3PL∕4wb2, in which P represents the load breaking the bar in newtons; L represents the distance between the support bars from center to center in millimeters; w indicates the width of the bar in millimeters; and b indicates to the thickness of the bar in millimeters.

### Cyclic loading and residual flexural strength test

The remaining specimens (*n* = 10) were subjected to mechanical aging through cyclic loading in a custom-built ACTA-type pneumatic fatigue tester. Cyclic loading was conducted in distilled water at 37 °C to simulate the intraoral hydrolytic environment. Each specimen underwent 2 million loading cycles at a frequency of 2 Hz using a stainless-steel spherical piston (6 mm diameter). The applied load corresponded to 63% of the average initial flexural strength of each treatment group, ensuring subcritical fatigue without inducing catastrophic failure [29–31]. The piston was routinely inspected for surface wear and replaced as needed to maintain consistent contact mechanics [29]. A 0.5 mm thick polyethylene sheet was interposed between the piston and specimen to serve as a stress-distribution interface and to minimize localized surface damage. Each cycle involved 5 s of contact in water to mimic clinical chewing conditions. Following ISO 6872:2015 [29], thermocycling was intentionally excluded to isolate the effects of mechanical fatigue from those of thermal aging. This approach aligned with standardized protocols for assessing flexural performance under repetitive loading while eliminating the influence of thermal stresses introduced. Following cyclic loading, specimens were subjected to monotonic loading to failure using the four-point flexural strength test protocol described in Sect. 1.4 to determine residual strength.

### Microstructure

Additional five specimens from each conventional and speed group were thermally etched at 1250 °C (to demonstrate the grain boundary network) and were gold sputter coated for evaluating for their microstructure. Also, for each surface treated group, three specimens were gold sputter coated and examined under scanning electron microscope (JEOL JSM-6400, Tokyo, Japan) to detect the surface criteria (*n* = 5 per group). Examination was performed at an accelerating voltage of 20 kV, and current emission of 10 µA. 1400 grains of zirconia were measured using Image-Pro PLUS 6.0 software (Media Cybernetics, Rockville Pike, USA). By applying the linear intercept approach (Fiji-ImageJ, National Institutes of Health, United States), the average grain sizes and standard deviations were obtained.

### Statistical analysis

With 40 specimens per group, a 0.40 effect size, and a 0.05 alpha error, the selected test of choice had adequate power (0.95) to detect statistical differences between the test groups (G*power software 3.1.9.7) [32, 33]. Weibull analysis (Weibull, 1951) was used to apply reliability statistics to the initial and residual flexural strength data. The characteristic strength was calculated based on a 63% failure value [30, 34]. Normality was checked for all variables using Shapiro wilk test and Q-Q plots. The Weibull modulus (m) was determined following the guidelines of DIN ENV 843-5 (2007) [35]. Data was analyzed using multivariate analysis of variance (ANOVA) and Bonferroni post-hoc test for pair-wise comparison (SPSS 22, IBM Inc., USA). The average grain size was statistically analyzed using the Krusal–Wallis test, Dunn’s test, and the Shapiro–Wilk test (*p* < 0.001).

## Results

### Initial and residual flexural strength test and Weibull analysis results

**Fig. 2:**
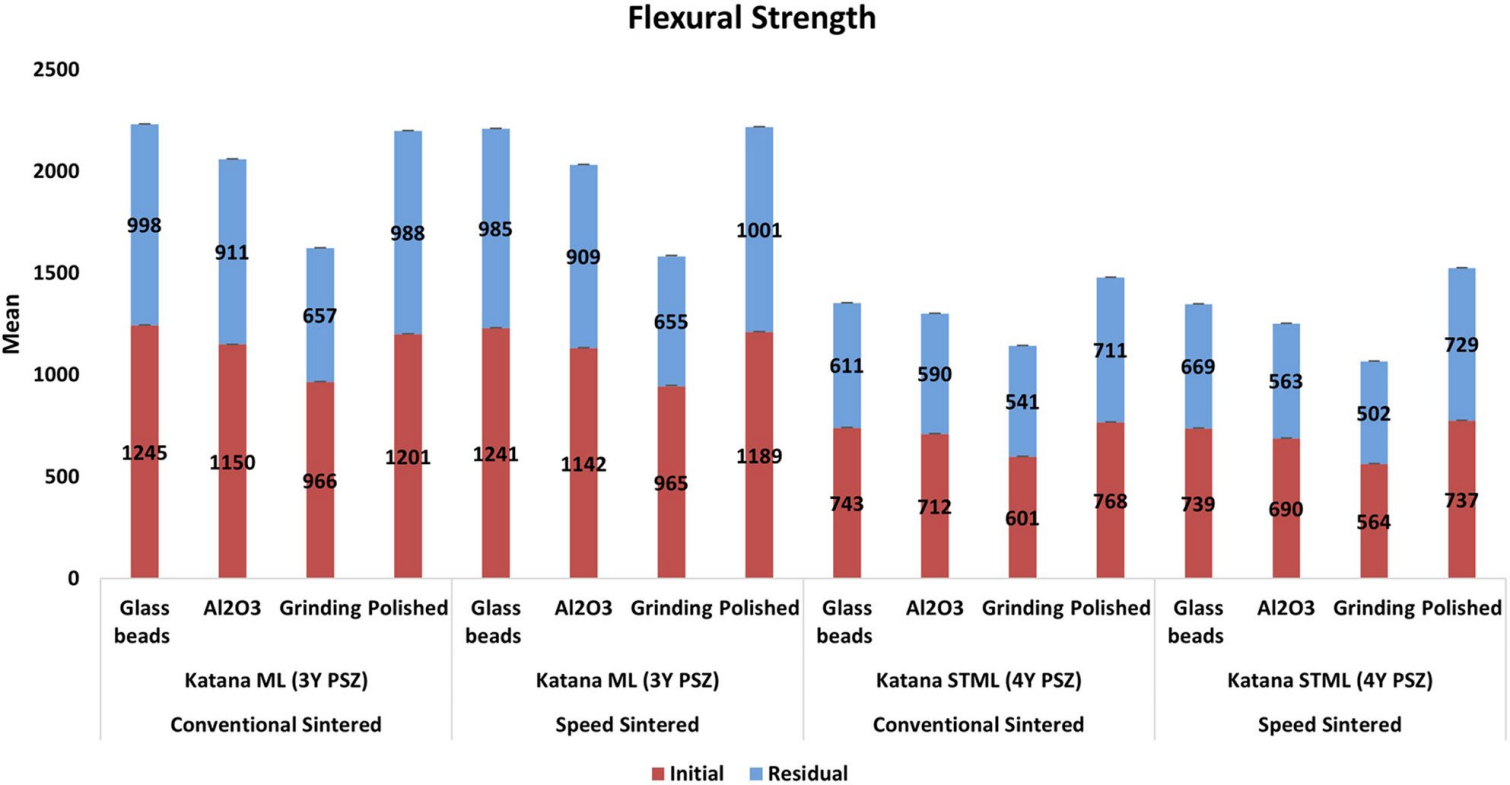
Initial and residual flexural strength of all tested subgroups

All tested variables had a significant effect on both initial and residual flexural strength of the tested materials. Significant effects were observed for zirconia type (*p* < 0.001), surface treatment (*p* < 0.001), sintering speed (*p* < 0.001), and cyclic loading (F = 31.1; *P* < 0.001) (Table 3). Speed sintering did not significantly influence the flexural strength of 3-YPSZ (*p* = 0.1), but it resulted in a statistically significant reduction in 4Y-PSZ (*p* < 0.001). 3-YPSZ maintained stable flexural strength following cyclic loading, with no statistically significant reduction (*p* = 0.082), whereas 4Y-PSZ demonstrated a significant decrease in flexural strength following cyclic loading (*p* < 0.001) (Table 4; Fig. 2). In 3Y-PSZ, glass bead airborne-particle abrasion produced the highest initial flexural strength values under both conventional and speed sintering conditions (*p* < 0.001). For 4Y-PSZ, the highest initial strength values were observed in the speed-sintered polished group (*p* < 0.001). In both zirconia types, grinding resulted in the lowest strength values, with the greatest reduction occurring in 4Y-PSZ.

**Table 3:** Multivariate analysis of variance ANOVA assessing the effect of zirconia materials, surface treatment, Cyclic loading and sintering on flexural strength

	Variables	F test	*P* value	Partial eta Squared
Flexural strength	Type of zirconia	163.1	*P* < 0.001*	0.98
	Sintering procedure	58.6	*P* < 0.001*	0.83
	surface treatment	88.4	*P* < 0.001*	0.89
	Cyclic loading	31.1	*P* < 0.001*	0.74
	Interaction surface treatment x Sintering procedure	180.2	*P* < 0.001*	0.99
	Interaction cyclic loading x Sintering procedure	120.4	*P* < 0.001*	0.96

*Statistically significant at *p* value ≤ 0.05, flexural strength Model Summary: Adjusted R squared = 0.999, *p* value < 0.0001

**Table 4:** Weibull modulus, initial and residual flexural strength of all tested subgroups

Conventional sintering							Speed sintering				
Group	Surface treatment	Flexural strength (Mpa) Mean ± SD		Weibull modulus		Reduction in strength percentage	Flexural strength (Mpa) Mean ± SD		Weibull modulus		Reduction in strength percentage
		Initial flexural strength	Residual flexural strength	Initial flexural strength	Residual flexural strength		Initial flexural strength	Residual flexural strength	Initial flexural strength	Residual flexural strength	
Katana ML (3Y PSZ)	air abrasion with glass beads	1245 ± 1.3^a^	998 ± 1.5^a^	12.9 ± 0.8^a^	12.8 ± 0.6^a^	19.87%	1241 ± 1.7^a^	985 ± 1.6^a^	12.6 ± 0.5^a^	12.5 ± 0.2^a^	20.47%
	air abrasion with Al_2_O_3_	1150 ± 1.1^b^	911 ± 1.3^b^	11.6 ± 0.5^b^	10.1 ± 0.8^b^	20.74%	1142 ± 1.6^b^	909 ± 1.8^b^	11.5 ± 0.3^b^	10.2 ± 0.5^b^	20.32%
	Grinding	966 ± 1.2^c^	657 ± 1.7^c^	9.1 ± 0.4^c^	7.2 ± 0.7 ^c^	31.99%	965 ± 1.8^a^	655 ± 1.7^a^	8.9 ± 0.6^a^	6.9 ± 0.4^a^	32.38%
	Polished	1201 ± 1.4^b^	988 ± 1.4 ^b^	12.7 ± 0.7^b^	11.5 ± 0.6^b^	17.73%	1189 ± 1.8^b^	1001 ± 1.4^b^	12.6 ± 0.7^b^	11.15 ± 0.3^b^	16.85%
Katana STML (4Y PSZ)	air abrasion with glass beads	743 ± 1.1^a, A^	611 ± 1.7^a, A^	12.1 ± 0.9^a, A^	11.3 ± 0.2^a, A^	17.77%	739 ± 1.9^c, A^	609 ± 1.5^c, A^	11.5 ± 0.5^c, A^	10.1 ± 0.3^c, A^	17.38%
	air abrasion with Al_2_O_3_	712 ± 1.3^b, A^	590 ± 1.5 ^b, A^	11.6 ± 0.5 ^b, A^	9.8.6 ± 0.7 ^b, A^	17.13%	690 ± 1.6^b, A^	563 ± 1.6^b, A^	11.2 ± 0.4 ^b, A^	8.3 ± 0.3 ^b, A^	18.41%
	Grinding	601 ± 1.2^c, B^	541 ± 1.7^c, B^	8.6 ± 0.7^c, B^	5.6 ± 0.5 ^c, B^	9.98%	564 ± 1.7 ^c, B^	502 ± 1.7 ^c, B^	8.6 ± 0.3 ^c, B^	5.1 ± 0.2 ^c, B^	10.99%
	Polished	768 ± 1.5^b, C^	711 ± 1.4^b, C^	12.8 ± 0.8^b, C^	11.4 ± 0.5 ^b, C^	7.42%	737 ± 1.8 ^b, C^	729 ± 1.5 ^b, C^	12.8 ± 0.4 ^b, C^	12.4 ± 0.4 ^b, C^	3.6%

Various superscript letters indicate statistically significant differences between surface treatment groups. (uppercase horizontal comparison, lowercase vertical comparison) (*p* < 0.05)

**Fig. 3:**
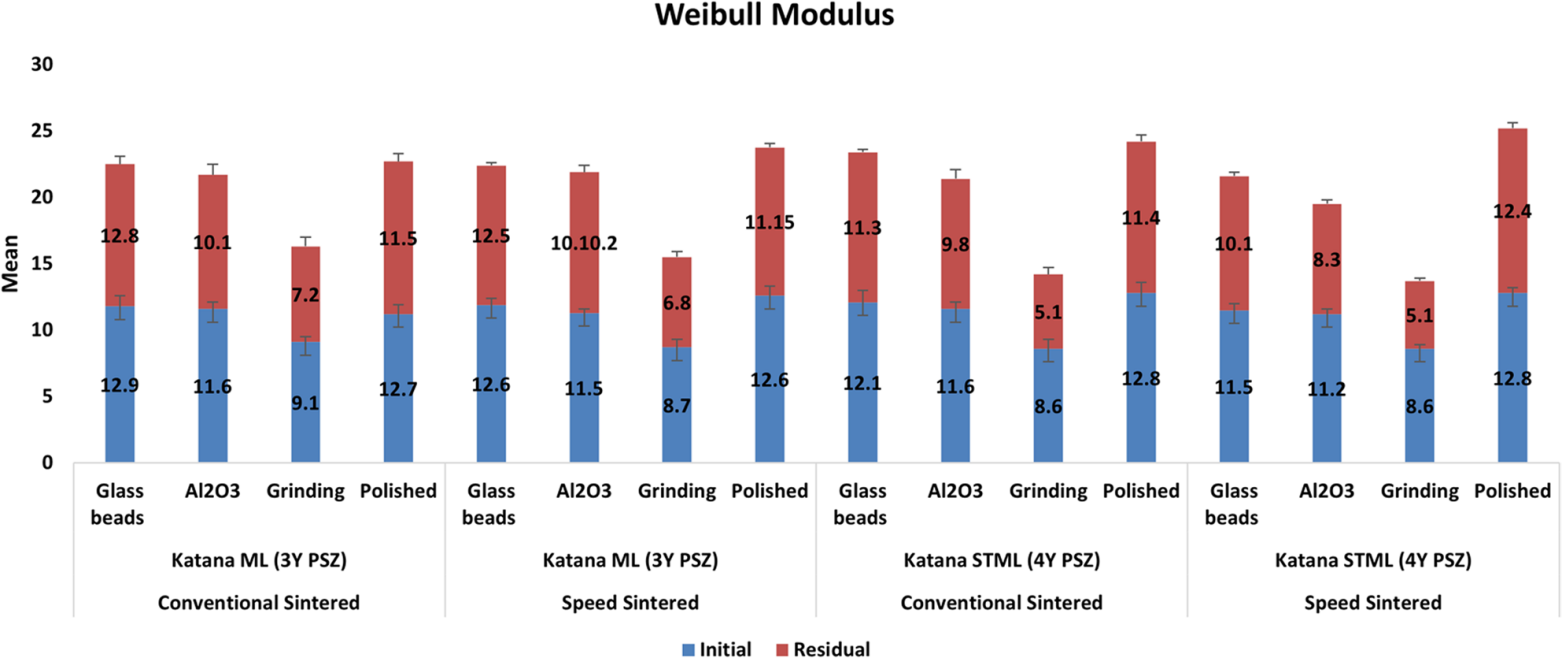
Weibull modulus of all tested subgroups

Glass bead-treated 3Y-PSZ exhibited the highest Weibull modulus (12.9), indicating superior reliability and resistance to flaw propagation. Similarly, polished 4Y-PSZ demonstrated the highest Weibull modulus (12.8), reflecting consistent mechanical performance. No significant differences were observed between glass bead and alumina air abrasion (*p* ≥ 0.41), suggesting comparable reliability between these treatments. Complete values for mean flexural strength, standard deviation, Weibull modulus, and percentage strength reduction are presented in (Table 4; Fig. 3).

The percentage reduction in flexural strength (Table 4) reflects the material’s susceptibility to cyclic loading and surface damage. The smallest reduction was observed in polished 4Y-PSZ under speed sintering (3.6%), indicating high surface integrity and reliability. In contrast, ground 3Y-PSZ and 4Y-PSZ exhibited reductions exceeding 30% and 10%, respectively, highlighting the detrimental impact of aggressive mechanical surface preparation. Glass bead treated 3Y-PSZ maintained a relatively low reduction (~ 20%), whereas alumina abrasion produced comparable or slightly greater losses, confirming the influence of abrasive particle hardness and induced flaw severity on long-term strength.

### Microstructure

**Fig. 4:**
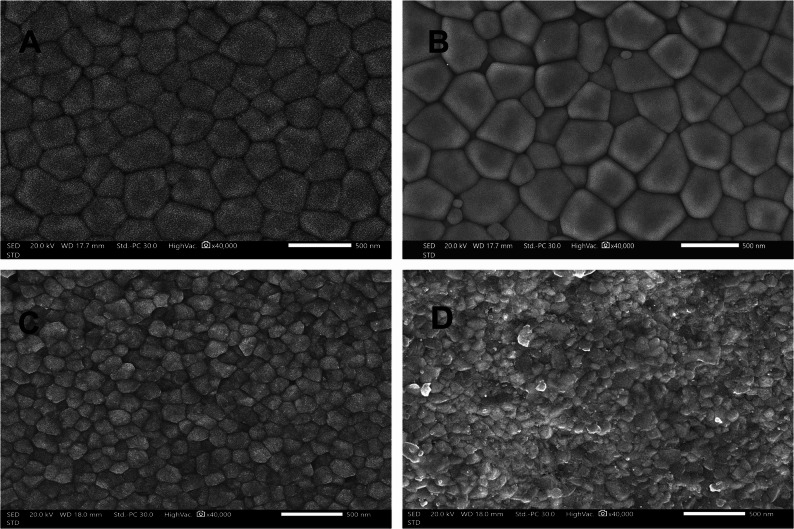
Scanning electron microscopic images (high vacuum, 20 kV, original magnification ×40,000; scale bar = 500 nm). **A**, conventionally sintered 4 mol% yttria partially stabilized zirconia (4-YPSZ). **B**, Speed sintered 4 mol% yttria partially stabilized zirconia (4-YPSZ) with unequal grain size distribution. **C**, Conventional 3Y-PSZ showed smooth surface densely packed relatively close in average grain size with well-defined and continuous boundaries and minimal porosity. **D**, Speed-sintered 3Y-PSZ maintained microscopic structure with a smaller average grain-size distribution and slightly broader with heterogeneously distributed fine grains with presence fine porosity

Two-way ANOVA of SEM image analysis showed that mean grain size was significantly influenced by both zirconia material composition and sintering protocol (*p* < 0.001). For 3Y-PSZ, conventional sintering produced an average grain size of approximately 0.94 μm, while speed sintering yielded a slightly smaller average grain size of about 0.79 μm. This difference in mean grain size between the sintering methods was statistically significant (*p* = 0.001). The grain size distributions for 3Y-PSZ overlapped considerably between the two methods, with measured grain sizes ranging from 0.6 to 1.7 μm in both groups (Table 5). For 4Y-PSZ, the grain growth was more markedly affected by the sintering protocol. Conventionally sintered 4Y-PSZ exhibited a larger mean grain size of 1.51 μm compared to speed-sintered 4Y-PSZ approximately 1.02 μm. This represented a substantial reduction in grain size with the speed sintering procedure, and the difference was highly significant (*p* < 0.001). Notably, conventionally sintered 4Y-PSZ also showed a much wider grain size range (0.54–2.95 μm), indicating the presence of some very large grains (and a few very small ones), whereas speed sintering limited the grain size to a narrower range (0.96–1.96 μm) (Table 5). At ×40,000 magnification, conventionally sintered 3Y-PSZ (Fig. 4A) showed a homogeneous microstructure of small, uniform grains with well-defined and tightly packed grain boundaries and minimal porosity. Speed-sintered 3Y-PSZ maintained overall structural integrity, changes in boundary metrics with localized grain boundary irregularities with presence of shallow intergranular porosity between oversized pull-out grains along boundaries (Fig. 4B). Conventionally sintered 4Y-PSZ (Fig. 4C) showed a smoother microstructure with larger grains, and well-defined boundaries compared to 3Y-PSZ. In contrast, speed-sintered 4Y-PSZ (Fig. 4D) exhibited additional grain coarsening, moderate grain pull-out, and less distinct grain boundaries, with interrupted fine-grain interrupted between large grains indicating obvious microstructural changes induced by accelerated sintering.

**Table 5:** Mean and standard deviation of grain in (µm) between conventional and speed sintering of 3-YPSZ and 4-YPSZ

		3-YPSZ	4-YPSZ	*P* value
Conventional Sintering	Average grain size	0.94 ± 0.66^a^	1.51 ± 0.87^b^	0.001*
	Min - Max	0.62–1.72	0.54–2.95	
Speed Sintering	Average grain size	0.79 ± 0.09^a^	1.02 ± 0.14^b^	< 0.001*
	Min - Max	0.48–1.67	0.96–1.96	
***P*** **value**		0.001*	< 0.001*	

*Statistically significant at *p* value ≤ 0.05, different superscript letters denote statistically significant difference between two groups

On 4Y-PSZ, airborne-particle abrasion with glass beads produced a heterogeneous, discontinuous topography with multiple angular residues embedded in the outermost surface, visible as bright, flake-like inclusions. By contrast, 3Y-PSZ exhibited a more uniform microrelief composed of compact, smoothly contoured grains and shallow hemispherical marks, with only scattered, rounded bright indentations consistent with smaller residual glass beads. Among the tested treatments, glass-bead abrasion yielded the lowest roughness and fewest angular surface irregularities.

**Fig. 5:**
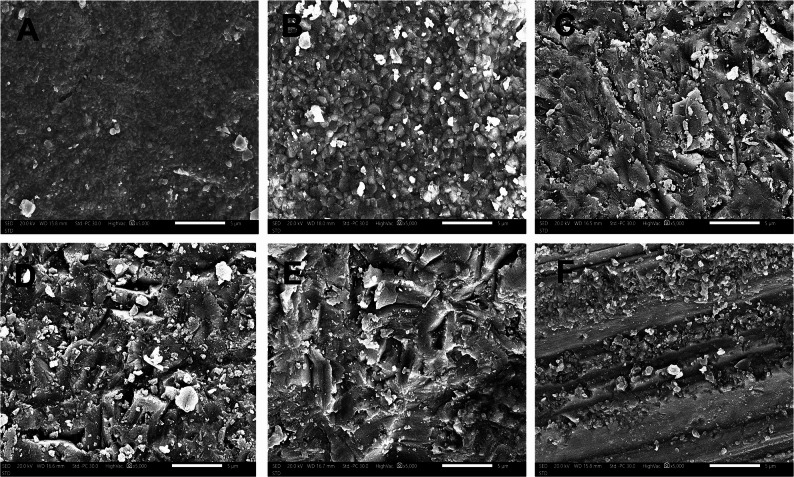
Scanning electron micrographs acquired with SED at 20 kV, working distance 16–18 mm; magnification ×5000; scale bar = 5 μm. **A**,3Y-PSZ after airborne-particle abrasion with glass beads; slightly distribution of abrasive residues. **B**,4Y-PSZ after airborne-particle abrasion with glass beads. The surface exhibited angular surface irregularities with flake-like delamination, and discrete bright, embedded glass particles. **C**,3Y-PSZ after airborne-particle abrasion with 50 μm Al₂O₃ with heterogeneous roughness, with angular surface irregularities with lamellar clustered alumina fragments, embedded alumina particulates visible as bright inclusions within the outer surface. **D**,4Y-PSZ after airborne-particle abrasion with 50 μm Al₂O₃, broader micro-chipping along edges; larger angular debris clusters than in 3Y-PSZ. **E**,3Y-PSZ after grinding; deep directional striations with smeared lamellae and particulate debris aligned to grinding direction. **F**,4Y-PSZ after grinding; well-oriented grooves with intermittent chip pull-outs; fewer large debris aggregated than after alumina abrasion

Airborne-particle abrasion with alumina generated substantially higher roughness and more pronounced micro-irregularities (Fig. 5C, D). In 3Y-PSZ, the surface showed angular grooves, lamellar (flake-like) delamination along grain boundaries, and discrete embedded alumina particles dispersed across the surface. 4Y-PSZ presented a more discontinuous, step-like relief characterized by broader lamellar detachments, and larger alumina remnants, producing sharper discontinuities at flake margins. The ground specimens exhibited the most irregular surface morphology, characterized by deep, parallel grooves, step-like ridges, and disrupted surface continuity consistent with aggressive mechanical material removal. These features were present in both 3Y-PSZ and 4Y-PSZ (Fig. 5E, F) and were more pronounced in 4Y-PSZ than in 3Y-PSZ, with broader groove spacing and sharper ridge breakage.

## Discussion

Following cyclic loading, the flexural strength of 4Y-PSZ was significantly affected by sintering speed, whereas 3Y-PSZ exhibited no significant changes under the same conditions. These findings partially rejected the null hypothesis and highlighted the distinct mechanical behavior of each zirconia type. Additionally, the influence of surface treatment protocols was evident across both materials, confirming the critical role of surface conditioning in determining the mechanical performance of monolithic zirconia restorations.

Among the tested surface protocols, glass bead airborne-particle abrasion significantly improved the flexural strength of 3Y-PSZ compared to the polished control. This enhancement is attributed to the activation of the tetragonal-to-monoclinic (t→m) phase transformation a recognized toughening mechanism without causing excessive surface damage [9, 18]. The compressive stresses generated during this transformation inhibit crack initiation and propagation. This finding supported the previous studies that have shown that low pressure particle abrasion had effectively balanced surface roughening and damage mitigation, particularly for 3Y-PSZ [18, 20]. In contrast, alumina airborne-particle abrasion and grinding resulted in a significant reduction in flexural strength for both 3Y-PSZ and 4Y-PSZ, with the impact being more pronounced in 4Y-PSZ. The increased hardness and particle size of alumina likely caused deeper surface flaws and microcracking that acted as stress concentration areas [20, 21]. Grinding introduced additional sharp-edged defects and potentially reversed beneficial phase transformations (m→t), increasing the material’s susceptibility to failure [20]. While some literature suggested that transformation toughening could partially compensate for surface damage, the current findings demonstrated that this mechanism alone was insufficient to counteract the mechanical degradation observed under these conditions [20, 22].

Polished 4Y-PSZ groups exhibited the highest Weibull modulus, signifying greater structural reliability and performance consistency. This suggested that surface integrity achieved through minimal intervention improved fracture resistance by limiting stress concentration areas. The lower transformation potential of 4Y-PSZ, due to its higher cubic-phase content, contributed to its reduced capacity for crack propagation, making it more susceptible to damage accumulation [9]. This was confirmed by SEM observations, among the tested surface treatments, glass-bead air abrasion yielded the smoothest zirconia surface topography, with the lowest roughness and minimal angular irregularities compared to polishing or grinding. These microstructural variations corelated with the observed finding differences in flexural performance. The gentle air abrasion action of spherical glass beads produced indentations and embedded silica into the zirconia surface, which could promote micromechanical interlocking and chemical adhesion via silane coupling, without significantly increasing roughness. This combination of low roughness and adequate texture is highly desirable for longevity of restoration. These findings agreed with previous studies on microstructural modifications induced by airborne abrasion and sintering conditions [18, 20, 22].

Cyclic loading, simulating seven years of of clinical use, had a stronger weakening effect on 4Y-PSZ than on 3Y-PSZ. While 3Y-PSZ maintained stable flexural strength after loading, 4Y-PSZ exhibited a significant decrease, suggesting superior fatigue resistance in 3Y-PSZ. This accounted for the variety in individual patient loading conditions, given that individuals typically perform 1000–2000 chewing cycles a day. Cyclic loading was isolated without thermocycling in accordance with ISO 6872:2015 to maintain the focus on load-induced effects and to make the study more clinically relevant [27, 29]. The crosshead speed of 1 mm/min was chosen in accordance with ISO 6872:2015 [29]. While intraoral loading rates vary and are typically slower in mastication (~ 1–2 Hz), faster crosshead speeds in testing helped to reduce environmental stress corrosion effects, thus reflecting intrinsic material strength [4]. Lower speeds increased subcritical crack growth, potentially reducing measured strength, whereas higher speeds may overestimate strength by minimizing crack propagation [4]. This standardized speed therefore represented a balance between clinical simulation and the need for reproducible, comparable laboratory results.

Sintering speed also showed contrasting effects between materials. Accelerated sintering did not significantly impact the flexural strength of 3Y-PSZ, likely due to its fine tetragonal grain structure that facilitated densification and minimized defect formation under rapid sintering protocols [14, 15]. In contrast, 4Y-PSZ experienced significant mechanical degradation, attributed to excessive grain growth, thermal stresses, and defect accumulation such as pores or intergranular voids. A previous study had reported the potential development of a rhombohedral phase under high-speed sintering, further compromising mechanical performance [17]. This discrepancy was primarily attributed to the distinct grain structures and phase compositions of the two zirconia types. In 4Y-PSZ, the presence of larger cubic grains limited complete densification and increased sensitivity to variations in sintering parameters. In contrast, the fine-grained tetragonal microstructure of 3Y-PSZ facilitated uniform densification, thereby maintaining mechanical stability under accelerated sintering protocols.

From a clinical perspective, within the limits of this in-vitro study, the higher fracture resistance of 3Y-PSZ favoured its use for high-load posterior restorations, including those with reduced thickness. In contrast, translucent zirconia such as 4Y-PSZ provided superior optical properties but exhibited greater susceptibility to surface damage and variability in mechanical performance, necessitating more conservative processing and handling.

The present study had limitations inherited to its in vitro design, and only two types of zirconia where used. Although cyclic loading was used to simulate long-term mechanical fatigue, the protocol did not reproduce the full complexity of the oral environment, including thermal cycling, enzymatic degradation, and different occlusal forces. In addition, only flexural strength and microstructural characteristics were assessed. Further studies on fracture toughness, wear resistance, and performance under combined thermomechanical and hydrothermal aging are needed to better estimate long-term behaviour. Studies should also include comprehensive material characterization. Quantitative phase analysis by X-ray diffraction can verify phase transformations caused by different sintering and surface treatments.

## Conclusions

Within the limitations of this in vitro study, the following conclusions were drawn:

1. Proper selection of surface treatment and sintering protocol is key to the long-term performance of various grades of zirconia restorations.
2. 3Y-PSZ proved more resilient to speed sintering and cyclic loading than 4Y-PSZ.
3. High Weibull Modulus associated with polished surfaces and glass bead particle abrasion, indicating minimal surface damage.
4. High-speed sintering can exacerbate the risk of failures of 4Y-PSZ zirconia due to preexisting of pores.

## Data Availability

The datasets used and/or analyzed during the current study available from the corresponding author on reasonable request.
